# In silico analysis of detrimental mutation in EPHB2 gene causing Alzheimer’s disease

**DOI:** 10.1186/1471-2164-15-S2-P46

**Published:** 2014-04-02

**Authors:** Iftikhar Aslam Tayubi, Sayane Shome, Omar M Barukab

**Affiliations:** 1 Faculty of Computing and Information Technology, King Abdulaziz University, Rabigh-21911, Saudi Arabia; 2 School of Biosciences and Technology,Vellore Institute of Technology,Vellore-632014, India

## Background

EPHB2 (Ephrin Receptor B2) are instrumental in signaling pathways like MAPK mediating tumour suppression, progenitor cell proliferation etc. [1,2]. Previous research has investigated the possibility that disrupted EphB2- NMDA R binding is relevant to the development of Alzheimer’s disease (AD), a condition that is characterized by severe synaptic impairment. Hence it can be inferred the role of EPHB2 and associated single nucleotide polymorphism is essential in studying the signaling pathways as well as neurodevelopmental processes associated with it. Studying the impact of the protein and the associated non-synonymous SNPS can also decode the role of genetic variations with respect to the role of the protein in the signaling pathway as well as susceptibility towards the disease.

## Materials and methods

**Figure 1:**
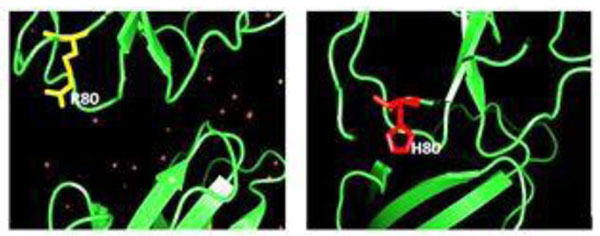
The native protein structure with Arginine (80) and mutant protein structure with Histidine (80) for SNP rs181872637. The mutant protein showed by SASA (Solvent Accessible Surfaces) and native protein showed lower values with time (Figure 2). The mutant protein shows deviations from RMSD values at the end of the time period.

The protein sequence data for EPHB2 [Accession ID: P29323.5] was collected from NCBI protein sequence database. SNP information for the computational analysis was obtained from NCBI dbSNP and Ensemble Gene Browser. Structure of EPHB2 protein was obtained from RCSB Protein Data Bank. (PDB ID:2QBX). A point mutation in native EPHB2 protein at position 80, Arginine (R) to Histidine (H) was introduced using SPDB Viewer package (Figure 1). We used SIFT, Polyphen, PhD-SNP and MutPred tools in order to examine the lethal nsSNPs occurring in the EPHB2 coding region. We filtered the most pathological mutation by combining the scores of then above servers. We implemented SNP Effect 4.0 to examine the possible phenotypic consequences at molecular, cellular, and individual level. Volume of the receptor binding cavity of native and mutant structure was examined by CASTp tool. Molecular dynamics simulation was performed by using Gromacs 4.5.3 package. Structure of native and mutant EPHB2 protein was used as starting point for MD simulations.

**Figure 2:**
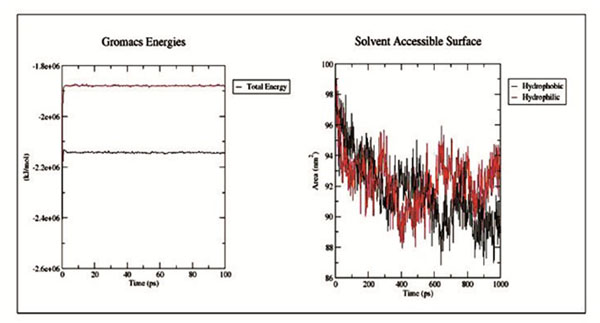
Total energy fluctuation in the native and mutant EPHB2 protein structure, native is shown in black and mutant in red.

## Results

**Figure 3:**
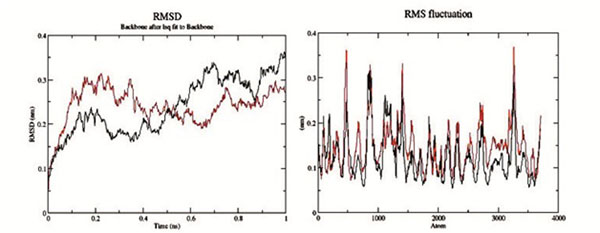
Backbone RMSDs and RMSF are determined as a function of time for native and Mutant EPHB2 protein at 300 K. Native is shown in black and mutant in red.

The mutation results in a ddG of 2.86 kcal/mol, accessible cavity reduced in mutant protein. Residues Ile196 and Tyr122 are no longer contributing to the stability of the protein. The mutant protein is unstable compared to the native protein (Figure 1):

The RMSF values suggest a higher degree of flexibility in the mutant compared to native protein (Figure 3).

## Conclusions

The mutation R80H is having adverse effects on the stability of the protein. It can be inferred from the results, the mutation affects the role of EPHB2. Further studies on its impact with its reactivity with NMDA receptor and role in the AD can be carried out.

## References

[B1] Cisse Reversing EphB2 depletion rescues cognitive functions in Alzheimer model Nature 2011 15 3 47 52 21113149 10.1038/nature09635 PMC3030448

[B2] Takasu Modulation of NMDA receptor-dependent Calcium influx and gene expression through EphB receptors Science 2002 15 5554 491 495 11799243 10.1126/science.1065983

